# Prognostic Value of MRI Anatomical Radiologic Scores for One-Year Neurological Outcome After Severe Traumatic Brain Injury

**DOI:** 10.1007/s12028-026-02474-7

**Published:** 2026-02-26

**Authors:** Théo Avignon, Vincent Doat-Sarfati, Thibault Agripnidis, Zacharie Cammilleri, Jean-François Hak, Jérémie Nakache, David Meunier, Julien Sein, Yanis Goutal-Guérin, Jeanne Morel, David Couret, Damien Galanaud, David Lagier, Lionel Velly, Alice Jacquens, Vincent Degos, Louis Puybasset, Pierre Simeone

**Affiliations:** 1 Department of Anesthesiology and Critical Care Medicine, Aix Marseille University, AP-HM, University Hospital Timone, Marseille, France; 2 Department of Anesthesiology and Critical Care Medicine, Sorbonne Université, AP-HP, Pitié-Salpêtrière Hospital, Paris, France; 3 Institut du Cerveau Et de La Moelle, Laboratoire GenoVasc, INSERM U1127, Paris, France; 4 Neuroimagery Department, Aix Marseille University, AP-HM, University Hospital Timone, Marseille, France; 5 Department of Public Health and Biostatistics, Aix-Marseille University, Marseille, France; 6 Timone Neuroscience Institute, CNRS, Aix Marseille University, Marseille, France; 7 Aix-Marseille University, CNRS, MRI- Center, INT, Institute of Neuroscience of la Timone, Marseille, France; 8 Department of Neuroradiology, Sorbonne Université, APHP, Pitié-Salpêtrière Hospital, Paris, France; 9 Neurocritical Care Unit, University Hospital Saint Pierre, Réunion Univ, Saint Pierre, La Réunion France; 10 Laboratoire d’Imagerie Biomédicale, Sorbonne Université, CNRS, INSERM, Paris, France; 11 Department of Anesthesiology and Critical Care Medicine, SAR1, Aix Marseille University, AP-HM, University Hospital Timone, Marseille, France

**Keywords:** Severe traumatic brain injury, Diffuse axonal injury, Magnetic resonance imaging, Prognosis, Radiologic scoring

## Abstract

**Background:**

Severe traumatic brain injury (sTBI) is a major cause of mortality and long-term disability, and early prognostic evaluation is essential to support therapeutic decision-making in intensive care. MRI is the reference imaging modality for detecting diffuse axonal injury (DAI), yet the prognostic accuracy and reproducibility of MRI-based radiologic scoring systems remain uncertain. This study aimed to compare the prognostic performance of five published MRI-based DAI scores for predicting 1-year neurological outcome, in a cohort of sTBI patients admitted to intensive care.

**Methods:**

We analyzed adult patients with sTBI included in the prospective MRI-COMA cohort (NCT00577954). Inclusion criteria were age ≥ 18 years, admission to ICU for sTBI, and absence of command following within 7 days after sedation withdrawal. Brain MRI was performed between day 7 and day 35 post-injury using standardized T1, FLAIR, T2*/SWI, and diffusion-weighted sequences. Three blinded evaluators (one neurointensivist, two neuroradiologists) independently applied five radiologic scores (Adams, Firsching, Hamdeh, Stockholm, Trondheim). The primary endpoint was the ability of each MRI-based radiologic score to predict 1-year neurological outcome, dichotomized as favorable (GOSE 5–8) or unfavorable (GOSE 1–4). Inter-rater reliability was quantified using Cohen’s and Fleiss’ kappa coefficients.

**Results:**

Between 2007 and 2023, 443 patients were screened and 185 met eligibility criteria. At one year, 111 patients (59%) had an unfavorable outcome. The prognostic performance of the five MRI scores was moderate, with AUCs ranging from 0.60 [CI95 0.52–0.68] to 0.70 [CI95 0.63–0.77], with no significant differences across scores or raters even between the intensivist and radiologists. Inter-rater reliability was fair to moderate (Fleiss’ κ = 0.28 [CI95 0.22–0.34] to 0.38 [CI95 0.33–0.43]).

**Conclusion:**

Conventional MRI-based scores have limited prognostic value and reproducibility in cases of sTBI and are therefore not suitable for accurate and objective neuroprognostication.

**Supplementary Information:**

The online version contains supplementary material available at 10.1007/s12028-026-02474-7.

## Introduction

Traumatic brain injury (TBI) remains a major public health concern due to its high incidence, mortality, and long-term disability burden. In France, approximately 150,000 patients are hospitalized each year for TBI, resulting in nearly 7,000 deaths [1]. Severe TBIs (sTBIs) account for up to 40% of all TBIs and require management in specialized neurocritical care units [2]. Among survivors, nearly half experience permanent disability, representing an estimated 345,000 people living with TBI-related impairments in France [3, 4].

Prognostic evaluation is essential in the acute phase of sTBI, as it directly influences therapeutic strategies, including decisions to continue or withdraw life-sustaining treatment. Inaccurate prognostication carries ethical and clinical implications — premature treatment withdrawal may occur in patients with potential for meaningful recovery, whereas continuation of care in cases of poor prognosis can lead to unnecessary suffering for patients, families, and caregivers. Indeed, nearly half of deaths in sTBI are associated with therapeutic limitations rather than fatal brain lesion [5].

Current prognostic tools rely on clinical and demographic parameters, such as the CRASH and IMPACT models [6, 7]. While useful at a population level, these models lack the accuracy required for individual decision-making. Other approaches, including electrophysiological monitoring or serum biomarkers (e.g., GFAP, UCH-L1), have shown variable predictive value [8]. In this context, neuroimaging—particularly magnetic resonance imaging (MRI)—has emerged as a promising modality for refining prognostic assessment.

Diffuse axonal injury (DAI) represents one of the most critical forms of secondary brain damage after TBI, resulting from acceleration–deceleration and rotational forces [9–11]. DAI typically affects the white matter tracts, especially at the gray–white matter junction, corpus callosum, and brainstem, and is strongly associated with unfavorable neurological outcomes [12]. Conventional computed tomography (CT) scanning, routinely performed in the acute phase, often fails to detect these microscopic lesions, whereas MRI remains the reference imaging technique for their identification [13–15]. Specific MRI sequences such as SWI, T2*, and diffusion-weighted imaging (DWI) allow the detection of both hemorrhagic and non-hemorrhagic DAI with greater sensitivity [16–19].

Several MRI-based radiologic scoring systems have been proposed to quantify the extent and topography of DAI and to predict neurological outcomes, including the Adams DAI score [20], Hamdeh score [21], Firshing classification [22], and the more recent Stockholm [23] and Trondheim scores [24]. However, previous studies are few, often retrospective, and methodologically heterogeneous, and no comparative evaluation of these scoring systems has been performed to date.

The present study aims to compare the prognostic performance of five MRI-based radiological scores to predict one-year neurological outcome in a cohort of patients with severe traumatic brain injury admitted to intensive care.

## Materials and Methods

### Study Population

This study analyzed brain MRI scans from patients enrolled at the Pitié–Salpêtrière Hospital (Paris, France) in the MRI-COMA cohort (NCT00577954) [25], a prospective, multicenter, observational study including comatose patients of various etiologies who underwent multimodal MRI. In the present analysis, only patients with sTBI admitted between 2007 and 2023 were included. Eligible patients were adults (> 18 years) admitted to intensive care units (ICU) with absent command following within 7 days following sedation withdrawal. Exclusion criteria included MRI contraindications, pre-existing neurological disease, life-threatening comorbidities, or major pre-injury disability.

### Ethical Approval and Consent

The study was approved by the local ethics committee (Comité de Protection des Personnes, CPP XI; authorization no. 1934708). In accordance with French law, patients or their legal representatives were informed about study participation, and informed consent was obtained before follow-up evaluations.

### Clinical Data and Follow-Up

Clinical, biological, and treatment-related data were prospectively collected during the first 14 days of intensive monitoring. Demographic, clinical, and biological data were extracted from electronic medical records, including medical history, comorbidities, medication use, ICU management, acute clinical presentation, secondary brain insults, laboratory parameters, and in-hospital complications or treatments. Neurological outcome was assessed one year after injury using the GOSE via structured telephone interviews with patients or relatives.

### MRI Acquisition

MRI examinations were performed between day 7 and day 35 post-admission on 1.5 T or 3 T scanners (GE Medical Systems, Siemens, or Philips). The protocol included a high-resolution 3D T1-weighted sequence (1 mm isotropic voxels), axial T2-FLAIR, axial T2* or SWI/SWAN gradient-echo sequences, DWI. Acquisition parameters varied slightly by manufacturer and are detailed in Supplementary Table 1.

### MRI Analysis

All MRIs were independently reviewed by three blinded raters—one neurointensivist and two neuroradiologists. Each rater identified DAI lesions and assigned grades for five published radiologic scoring systems: the Adams DAI score, Firsching score, Hamdeh score, Stockholm score, and Trondheim score. These scores are detailed in Supplementary Table 2. An example of how the grading was performed is provided in Supplementary Fig. 1.

### Outcome

The primary endpoint was the ability of each MRI-based radiologic score to predict 1-year neurological outcome, dichotomized as favorable (GOSE 5–8) or unfavorable (GOSE 1–4). The GOSE is detailed in the Supplementary Table 3. Secondary outcomes were the inter-rater variability in MRI-scores and the influence of evaluator's medical specialty on score robustness.

### Statistical Analysis

Continuous variables were expressed as mean ± SD or median (IQR), and categorical variables as n (%). Group comparisons used Chi-square or Fisher’s exact tests for categorical data and Student’s t-test or Wilcoxon tests for continuous data, as appropriate. Statistical analyses were performed using *R* (version 4.5.1) and Jamovi (version 2.6). Diagnostic performance (sensitivity, specificity, positive and negative predictive values) and receiver operating characteristic (ROC) curves with area under the curve (AUC) were computed using the *Statistics Kingdom* tool based on DeLong’s method [26]. Inter-rater reliability was assessed using Cohen’s linear weighted kappa for each rater pair and Fleiss’ kappa for overall agreement, interpreted according to standard thresholds (< 0.2 poor, > 0.8 almost perfect) [26].

## Results

### Patient Characteristics

**Fig. 1:**
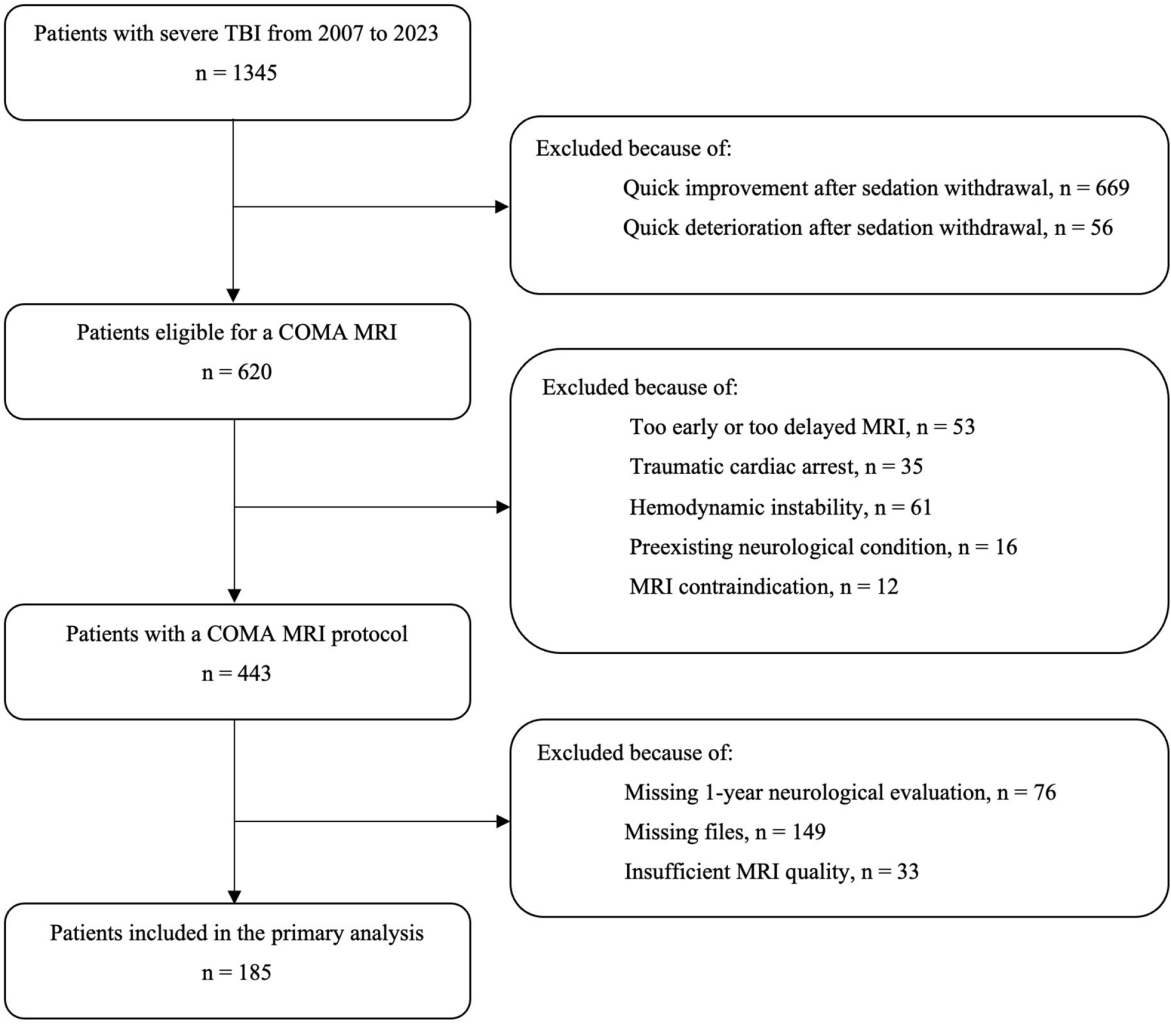
Flow chart

Among 443 initially screened patients, 185 with sTBI met all eligibility criteria and were included in the final analysis (Fig. 1). Among these patients, 111 (59%) had an unfavorable neurological outcome (GOSE 1–4), while 74 (41%) showed a favorable outcome (GOSE 5–8) at one year. The mean delay between admission and MRI acquisition was 22.9 ± 9.6 days and did not differ significantly between groups (Table 1). The mean age of the cohort was 39.9 ± 15.6 years. Patients with a favorable outcome were significantly younger than those with poor recovery (35.0 ± 13.2 vs. 43.2 ± 16.3 years, *p* = 0.0002). No significant differences were found between groups regarding comorbidities such as obesity, alcoholism, psychiatric disorders, or chronic neurodegenerative diseases. Among patients with an unfavorable outcome, 61 (55%) died in the ICU, accounting for 86% of all deaths in the cohort; no deaths occurred among patients with favorable recovery. Withdrawal of life-sustaining therapy occurred in 54 (27,8%) patients.

**Table 1:** Demographical data

	Global population (*n* = 185)	Good neurological outcome (GOSE 5–8, *n* = 74)	Poor neurological outcome (GOSE 1–4, *n* = 111)	*p*-value
Male sex, n (%)	152 (82)	62 (83)	90 (81)	0.78
Age, mean (±SD)	39.9 (15.6)	35 (13.2)	43.2 (16.3)	0.0002
Psychiatric disorders, n (%)	17 (11)	7 (9)	10 (9)	1
Drug abuse, n (%)	18 (12)	11 (15)	7 (6)	0.12
Neurological disease, n (%)	3 (2)	0	3 (3)	0.26
Alcohol abuse, n (%)	38 (26)	15 (20)	23 (21)	0.87
BMI > 30, n (%)	11 (7)	4 (5)	7 (6)	0.97
Antiplatelet therapy, n (%)	8 (5)	2 (3)	6 (5)	0.53
Anticoagulant therapy, n (%)	11 (7)	6 (8)	5 (5)	0.57
ICU mortality, n (%)	61 (36)	0	61 (55)	0.005
Time to MRI in days, mean (±SD)	22.9 (9.6)	24.8 (11.6)	21.9 (8)	0.06

*BMI* Body mass index, *ICU* Intensive care unit, *MRI* Magnetic resonance imaging

Regarding trauma characteristics, the only significant difference was the higher peak norepinephrine dose in the unfavorable outcome group (3.77 ± 3.70 vs. 2.53 ± 2.21 mg/h; *p* = 0.02). Orthopedic injuries were present in 87 (59%) and spinal injuries in 40 (27%) of cases. One hundred and twenty-five patients (87%) underwent intracranial pressure monitoring, 103 (72%) had an external ventricular drain, 29 (20%) received therapeutic hypothermia, and 13 (9%) underwent metabolic suppression. ICU and mechanical ventilation durations were longer in the poor-outcome group (ICU stay: 38.9 ± 19.9 vs. 32.2 ± 14.6 days, *p* = 0.03; ventilation: 25.1 ± 13.1 vs. 18.3 ± 10.8 days, *p* = 0.001). Decompressive craniectomy was also more frequent in this group (16 (14%) vs. 3 (4%), *p* = 0.05). All clinical data at admission and ICU management details are presented in Supplementary Tables 4 and 5.

### Prognostic Performance of MRI Scores

**Fig. 2:**
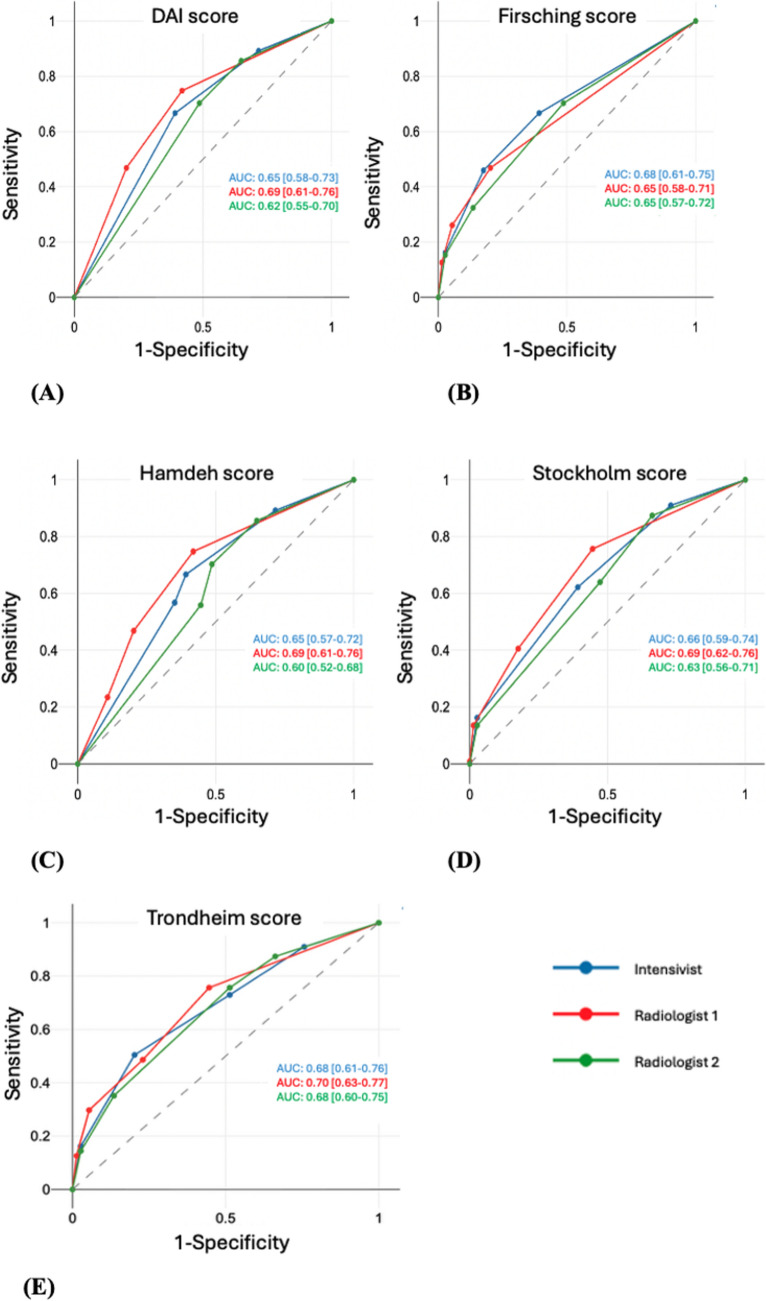
ROC curves and areas under the curve (AUCs) for each evaluator and each score. **A** DAI score, **B** Firsching score, **C** Hamdeh score, **D** Stockholm score, **E** Trondheim score

ROC analysis demonstrated similar prognostic performance among all five MRI-based scoring systems. The highest AUC was observed for the Trondheim score (AUC = 0.70 [0.63–0.77]), while the lowest was for the Hamdeh score (AUC = 0.60 [0.52–0.68]) (Fig. 2). No significant differences were found between AUCs across raters or scores (Supplementary Tables 6 and 7). Sensitivity, specificity, positive predictive value (PPV), and negative predictive value (NPV) are detailed in Supplementary Table 8. Median GOSE values decreased progressively with increasing MRI score grade, consistent across all raters, indicating that higher radiologic grades were associated with poorer neurological outcomes, although without statistically significant inter-rater variation (Supplementary Fig. 2 and Table 9).

### Inter-Rater Agreement

**Fig. 3:**
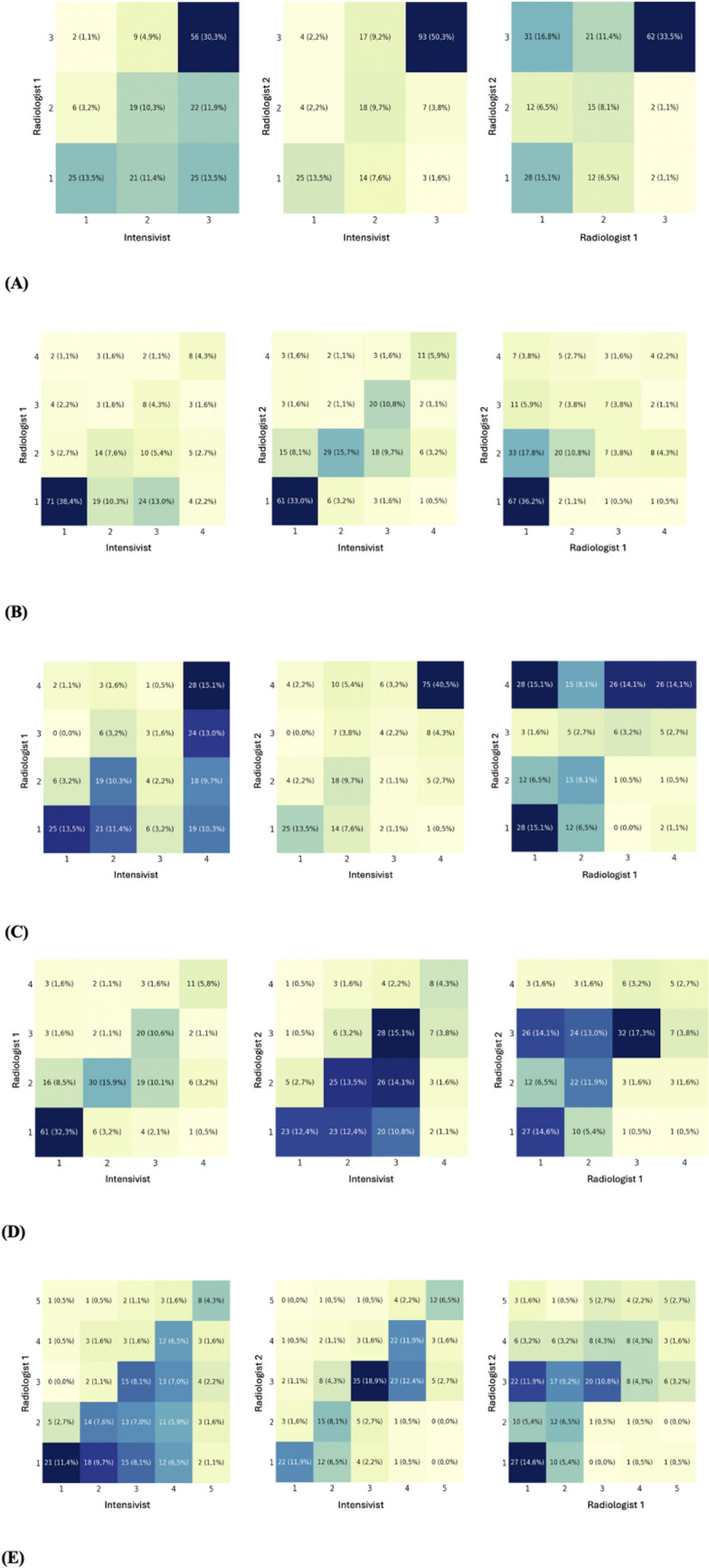
Confusion matrices showing the interpretations of the different raters for each scoring system with **A** DAI score, **B** Firsching score, **C** Hamdeh score, **D** Stockholm score and **E** Trondheim score

Inter-rater reliability between the three independent MRI evaluators was overall fair to moderate. Pairwise Cohen’s kappa values ranged from 0.31 to 0.63, with the highest agreement observed for the DAI and Hamdeh scores between the neurointensivist and one radiologist (κ = 0.63 [0.52–0.74]) and the lowest for the Hamdeh score between the two radiologists (κ = 0.31 [0.16–0.46]) (Table 2). Fleiss’ kappa confirmed these trends, with the strongest overall concordance for the DAI score (κ = 0.38 [0.33–0.43]) and the lowest for the Hamdeh score (κ = 0.28 [0.22–0.34]) (Table 2). Inter-observer agreement is illustrated in the confusion matrices in Fig. 3.

**Table 2:** Cohen’s kappa Coefficients and Fleiss’ kappa coefficient for inter-rater agreement according to radiological scores

Score	Specialists	Cohen’s kappa coefficient	Fleiss’ kappa coefficient
DAI	Intensivist–Radiologist 1	0.36 [0.25- 0.47]	0.38 [0.33–0.43]
	Intensivist–Radiologist 2	0.63 [0.52- 0.74]	
	Radiologist 1- Radiologist 2	0.38 [0.24- 0.51]	
Firsching	Intensivist–Radiologist 1	0.34 [0.19- 0.49]	0.36 [0.31–0.42]
	Intensivist–Radiologist 2	0.58 [0.46- 0.70]	
	Radiologist 1–Radiologist 2	0.33 [0.18- 0.47]	
Hamdeh	Intensivist–Radiologist 1	0.32 [0.17- 0.46]	0.28 [0.22–0.34]
	Intensivist–Radiologist 2	0.63 [0.52- 0.74]	
	Radiologist 1–Radiologist 2	0.31 [0.16- 0.46]	
Stockholm	Intensivist–Radiologist 1	0.34 [0.20- 0.48]	0.32 [0.27–0.38]
	Intensivist–Radiologist 2	0.61 [0.49- 0.73]	
	Radiologist 1–Radiologist 2	0.32 [0.18- 0.46]	
Trondheim	Intensivist–Radiologist 1	0.32 [0.19- 0.45]	0.29 [0.23–0.36]
	Intensivist–Radiologist 2	0.61 [0.49- 0.72]	
	Radiologist 1–Radiologist 2	0.34 [0.20- 0.48]	

Results are presented as κ [95% CI]. Cohen’s and Fleiss’ kappa coefficient were interpreted according to the Landis and Koch scale: 0.21–0.40 = fair to moderate agreement; 0.41–0.60 = moderate agreement; 0.61–0.80 = substantial agreement

## Discussion

To our knowledge, this is the first study to simultaneously compare five established MRI-based radiologic scores within the same cohort of sTBI patients, using multiple independent blinded raters and standardized long-term neurological outcomes. This comprehensive design aims to provide a robust and comparative assessment of their prognostic performance and reproducibility.

The primary finding of this study is that the prognostic performance of five MRI-based radiologic scores for predicting 1-year neurological outcomes in patients with sTBI was only moderate. Across all raters, the AUC ranged from 0.60 to 0.70, with no significant differences between scores or evaluators. These results indicate that, while MRI-based score can capture clinically relevant patterns of DAI, their discriminative power remain limited when they’re used with conventional MRI sequences. Similar findings were reported by Tjerkaski et al. [23], whereas Moen et al. [24] observed higher AUCs (0.80–0.84) under more standardized and earlier imaging conditions. The lower performance observed in our cohort may be explained by the delay in MRI acquisition (median 23 days versus 9 days in Moen et al. [24], or 8 days and 7 days for Firshing [22], Hamdeh [21] and Tjerkaski [23] respectively), which likely reduces lesion visibility as diffusion and susceptibility signals fade over time. In addition, the limited sensitivity of conventional MRI to detect non-hemorrhagic axonal lesions has probably contributed to the low prognostic accuracy.

Another explanation is that our cohort more closely reflects real-life prognosis issue, with patients whose trajectories are not clearly favorable or unfavorable but instead fall into a gray zone of diagnostic and prognostic uncertainty since these are only patients who have abnormal neurological examination results after sedation is stopped. In contrast, some studies sometimes include another type of patients —patients who either awaken rapidly or never awaken at all— and tend to artificially inflate the apparent predictive performance of prognostic scores by including patients for whom practitioners would have had greater certainty regarding prognosis without necessarily requiring additional tools. Our more heterogeneous and pragmatic sample therefore likely attenuates the predictive value of these scores, highlighting their limited external validity when applied to real-world patients.

Despite these limitations, some specific radiologic features retained strong prognostic significance. Bilateral pontine lesions, corresponding to the highest grades in several scoring systems, were consistently associated with poor outcomes and showed excellent specificity (0.97–0.99) [21, 23, 24]. This supports prior evidence emphasizing the critical role of brainstem integrity in the recovery of consciousness [27, 28]. However, given the variability in image interpretation, even such severe findings should be integrated cautiously into prognostic reasoning and therapeutic decision-making.

The second major result concerns inter-rater reliability, which was overall fair to moderate. Cohen’s kappa ranged from 0.31 to 0.63, and Fleiss’ kappa from 0.28 to 0.38. Only isolated rater pairs achieved good agreement for specific scores (DAI, Hamdeh, Stockholm, Trondheim). These results are lower than previously reported (κ up to 0.96 in some studies) [29], likely due to differences in reader expertise, study design, and the subjective nature of visual lesion assessment.

The fair to moderate inter-rater reliability observed in our study raises important clinical implications, as such variability may limit the use of these radiologic scores in multicenter research or routine clinical practice. The agreement was not higher between radiologists than between the neurointensivist and radiologists, suggesting that variability is driven more by the intrinsic subjectivity of visual MRI assessment than by reader expertise. Allowing evaluators to select sequences, while reflective of real-world conditions, may also have contributed to heterogeneity. Nevertheless, prognostic performance remained consistent across raters, indicating that these interpretative differences did not substantially affect overall predictive trends.

Our findings highlight the intrinsic limitations of conventional MRI for neuroprognostication in sTBI. Newer imaging modalities, particularly diffusion tensor imaging (DTI), offer more quantitative and sensitive assessment of white matter integrity. Several studies, including those from the MRI-COMA consortium, have demonstrated that DTI metrics such as Fractional Anisotropy (FA) and Mean Diffusivity (MD) correlate strongly with neurological outcomes. Puybasset et al. [25] proposed a composite model combining age, FA, and MD, achieving an AUC of 0.89 for predicting long-term recovery. These findings suggest that DTI could complement or even surpass morphological scoring systems by providing objective, reproducible biomarkers of axonal injury.

A major strength of this study is the large and well-characterized cohort, derived from a prospective, multicenter initiative with standardized clinical data collection and 1-year outcome assessment. The sample size is considerably larger than in most prior studies evaluating MRI-based prognostic markers in sTBI, which enhances the robustness and external validity of the findings. In addition, all MRIs were independently assessed by three blinded evaluators, allowing a rigorous examination of inter-rater reliability and the influence of reader background on scoring performance. Although one of the readers was a neurointensivist rather than a neuroradiologist, this did not appear to increase inter-rater variability, as agreement between the neurointensivist and the neuroradiologists was comparable to that observed between the two radiologists, suggesting that subjectivity is intrinsic to the visual interpretation of these lesions rather than dependent on specialty.

Several limitations should also be acknowledged. Raters were free to choose which MRI sequences to prioritize when applying the scores. While this approach mirrors real-world clinical conditions, it likely increases extrinsic variability in scoring and may limit reproducibility across centers. Additionally, the MRI examinations were performed relatively late after injury, at a mean of approximately three weeks post-trauma. At this stage, both hemorrhagic and non-hemorrhagic lesions may become less conspicuous due to signal evolution, potentially reducing the sensitivity and prognostic power of conventional MRI-based scores. Similarly, the interpretability and use of sequences for the topographic diagnosis of DAI were left to the discretion of the interpreter. This may have introduced bias, but also reflects real-world interpretation conditions. Finally, it cannot be ruled out that a self-fulfilling prophecy effect may have existed and that patients with the most severe images were more likely to undergo end-of-life procedures.

## Conclusion

In this cohort of patients with severe traumatic brain injury, MRI-based radiologic scores showed only moderate prognostic accuracy and fair inter-rater reliability. Conventional MRI, though useful for identifying structural lesions such as diffuse axonal injury, remains insufficient for reliable long-term neuroprognostication. Bilateral pontine lesions were highly specific for poor outcomes but inconsistently identified between raters. These findings emphasize the limitations of qualitative MRI scoring in guiding clinical decisions. Future multicenter validation of multimodal prognostic models integrating DTI and automated imaging analysis could help bridge the gap between neuroimaging research and clinical decision-making.

## Supplementary Information

Below is the link to the electronic supplementary material.

Supplementary file1 (DOCX 2092 KB)

## Data Availability

Anonymized data are available to qualified investigators on request for the purposes of replicating procedures or results by contacting the corresponding author.

## Abbreviations

AUC: Area under the curve
CT: Computed tomography
DAI: Diffuse axonal injury
DTI: Diffusion tensor imaging
DWI: Diffusion-weighted imaging
FA: Fractional anisotropy
GCS: Glasgow coma scale
GOSE: Glasgow outcome scale–extended
ICU: Intensive care unit
MD: Mean diffusivity
MRI: Magnetic resonnance imaging
NPV: Negative predictive value
PPV: Positive predictive value
ROC curve: Receiver operating characteristic curve
sTBI: Severe traumatic brain injury
TBI: Traumatic brain injury
