# Supplementary material for: Prognostic Value of MRI Anatomical Radiologic Scores for One-Year Neurological Outcome After Severe Traumatic Brain Injury
Source: Neurocrit Care. 2026 Feb 26;45(1):304–13. doi: 10.1007/s12028-026-02474-7 (PMC13369353; doi:10.1007/s12028-026-02474-7)
Supplement: Supplementary file 1 — Supplementary file1 (DOCX 2092 KB) [file 12028_2026_2474_MOESM1_ESM.docx]

**Prognostic Value of MRI Anatomical Radiologic Scores for one-Year Neurological Outcome after Severe Traumatic Brain Injury**

-

***Supplementaries***

Table of contents

[Supplementary Table 1. MRI acquisition parameters for each scanner 3](#_Toc213324146)

[Supplementary Table 2. Radiological scores 4](#_Toc213324147)

[Supplementary Figure 1. Illustration of diffuse axonal injury quantification on MRI 6](#_Toc213324148)

[Supplementary Table 3. Extended Glasgow Outcome Scale (GOSE) 7](#_Toc213324149)

[Supplementary Table 4. Clinical data at ICU admission 8](#_Toc213324150)

[Supplementary Table 5. Specific management in ICU 9](#_Toc213324151)

[Supplementary Table 6. AUC according to each scoring system and each rater 10](#_Toc213324152)

[Supplementary Table 7. Results of AUC comparison tests between intensivists and radiologists 11](#_Toc213324153)

[Supplementary Table 8. Sensitivity, specificity, PPV, and NPV analyses for each scoring system according to each rater 12](#_Toc213324154)

[Supplementary Figure 2. Distribution of GOSE scores by grade and by rater for each radiological scoring system 1](#_Toc213324155)

[Supplementary Table 9. Median GOSE values for each scoring system and each grade according to each rater 10](#_Toc213324156)

[References 11](#_Toc213324157)

# Supplementary Table 1. ****MRI acquisition parameters for each scanner****


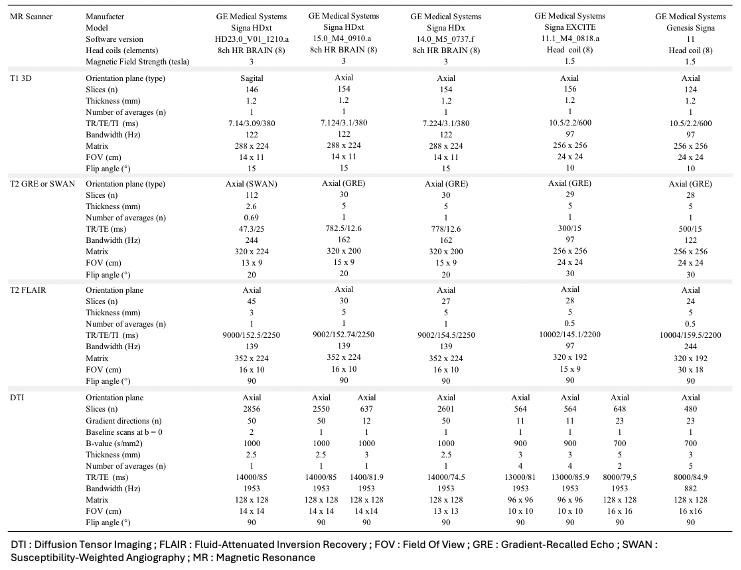


# Supplementary Table 2. Radiological scores

| DAI Score (1) | Radiological description |
| --- | --- |
| Grade 1 | Lobar white matter lesions only |
| Grade 2 | Corpus callosum lesions |
| Grade 3 | Brainstem lesions |

| Firsching Score (2) | Radiological description |
| --- | --- |
| Grade 1 | Supratentorial lesions only, without brainstem involvement |
| Grade 2 | Unilateral brainstem lesions at any level, with or without grade 1 lesions |
| Grade 3 | Bilateral midbrain lesions, with or without grade 2 lesions |
| Grade 4 | Bilateral pontine lesions, with or without grade 3 lesions |

| Hamdeh Score (3) | Radiological description |
| --- | --- |
| Grade 1 | Hemispheric lesions |
| Grade 2 | Corpus callosum lesions |
| Grade 3 | Brainstem lesions |
| Grade 4 | Substantia nigra or mesencephalic tegmentum lesions |

| Stockholm Score (4) | Radiological description |
| --- | --- |
| Grade 1 | All patients not fulfilling the criteria for grades 2 to 4 |
| Grade 2 | Unilateral thalamic or pontine lesions, and/or unilateral or bilateral lesions of the corpus callosum or midbrain outside the tegmentum |
| Grade 3 | Mesencephalic tegmentum lesions (unilateral or bilateral) and/or bilateral thalamic lesions and/or lesions of the posterior limb of the internal capsule |
| Grade 4 | Bilateral pontine lesions |

| Trondheim Score (5) | Radiological description |
| --- | --- |
| Grade 1 | Lobar or cerebellar white matter lesions |
| Grade 2 | Corpus callosum lesions |
| Grade 3 | Unilateral brainstem or thalamic lesions, or unilateral/bilateral basal ganglia lesions |
| Grade 4 | Bilateral midbrain or thalamic lesions |
| Grade 5 | Bilateral pontine lesions |

Supplementary Figure 1. Illustration of diffuse axonal injury quantification on MRI
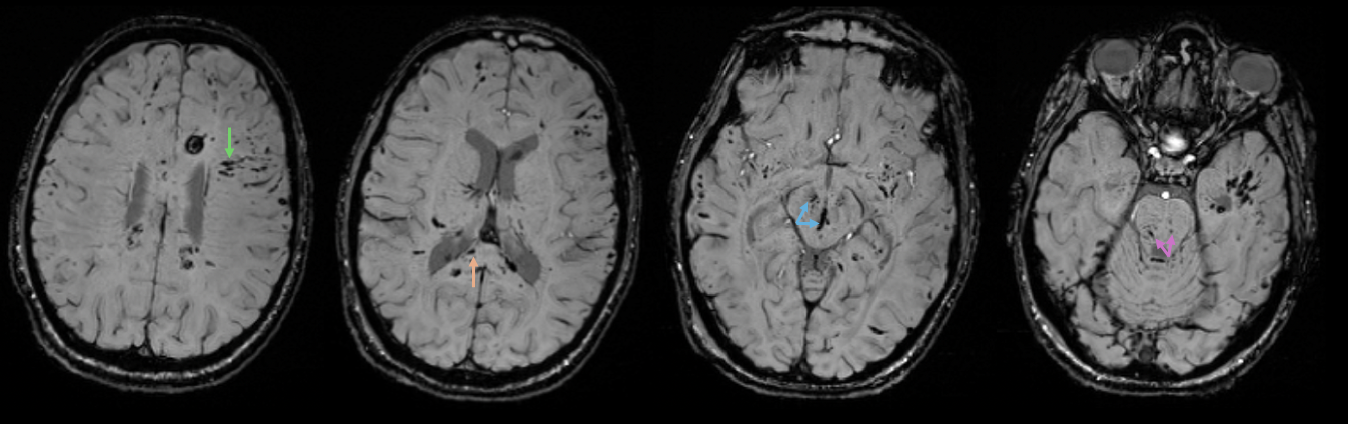


*Axial SWI images at different brain levels show multiple diffuse axonal injuries (DAI) appearing as hypointense foci: in the hemispheric lobar white matter (green arrow), the corpus callosum (orange arrow), and bilaterally in the midbrain (blue arrow) and pons (pink arrow). Radiological grading by the three evaluators was concordant: grade 3 on the DAI Score, grade 4 on the Firsching scale, grade 4 on the Hamdeh scale, grade 4 on the Stockholm scale, and grade 5 on the Trondheim scale.*

# Supplementary Table 3. Extended Glasgow Outcome Scale (GOSE)

**The GOSE** is a global functional outcome scale comprising eight categories. It subdivides the original categories of severe disability, moderate disability, and good recovery into upper and lower levels.

| Score | Level | Description |
| --- | --- | --- |
| 1 | Death | Patient deceased |
| 2 | Vegetative state | Patient alive but without conscious interaction with the environment |
| 3 | Severe disability, lower level (fully dependent) | Requires daily assistance for all activities |
| 4 | Severe disability, upper level (partially dependent) | Able to perform some activities of daily living independently, but requires assistance for others |
| 5 | Moderate disability, lower level (some dependence) | Independent in most activities of daily living, but limited in social or occupational functioning |
| 6 | Moderate disability, upper level (independent at home) | Independent, but with restrictions in certain complex or social activities |
| 7 | Good recovery, lower level (with residual limitations) | Able to resume almost all activities, with only mild limitations |
| 8 | Good recovery, upper level (return to normal life) | No significant functional deficits ; full retunr to pre-injury activities |

# Supplementary Table 4. Clinical data at ICU admission

|  | **Global population**  **(n = 185)** | **Good neurological outcome**  **(GOSE 5-8, n = 74)** | **Poor neurological outcome**  **(GOSE 1-4, n = 111)** | **p-value** |
| --- | --- | --- | --- | --- |
| **Hypotension,**  **n (%)** | 25 (15) | 8 (11) | 17 (15) | 0.59 |
| **Use of vasopressors.**  **n (%)** | 109 (76) | 44 (59) | 65 (59) | 0.34 |
| **Maximum dose of norepinephrine dose on admission in mg/h.**  **mean (± SD)** | 3.27 (3.20) | 2.53 (2.21) | 3.77 (3.70) | **0.02** |
| **Hemoglobin on admission.**  **mean (± SD)** | 12.5 (6.9) | 12.3 (2.2) | 12.6 (8.7) | 0.75 |
| **pH on admission.**  **mean (± SD)** | 7.28 (0.50) | 7.33 (0.11) | 7.25 (0.64) | 0.32 |
| **Lactate level on admission.**  **mean (± SD)** | 2.6 (2.2) | 2.7 (2.4) | 2.6 (2.0) | 0.56 |
| **Hypoxemia.**  **n (%)** | 33 (19) | 14 (19) | 19 (17) | 0.85 |
| **ARDS.**  **n (%)** | 57 (40) | 27 (36) | 30 (27) | 0.5 |
| **Hypocapnia.**  **n (%)** | 19 (14) | 11 (15) | 8 (7) | 0.21 |
| **Hypercapnia.**  **n (%)** | 19 (14) | 11 (15) | 8 (7) | 0.21 |
| **Blood glucose.**  **mean (± SD)** | 8.4 (3.9) | 7.7 (3.9) | 8.8 (3.8) | 0.08 |
| **Presence of myoclonic epilepsy.**  **n (%)** | 1 (1) | 0 | 1 (1) | 1 |
| **Seizure during ICU stay.**  **n (%)** | 7 (5) | 3 (4) | 4 (4) | 1 |
| **Mydriasis. n (%)**   - **Absent** - **Unilateral** - **Bilateral** | 96 (54)  36 (20)  46 (26) | 43 (58)  12 (16)  14 (19) | 51 (46)  24 (22)  32 (29) | 0.11  0.36  0.13 |
| **Extracranial orthopedic trauma.**  **n (%)** | 87 (59) | 36 (49) | 51 (46) | 0.94 |
| **Extracranial spinal trauma.**  **n (%)** | 40 (27) | 20 (27) | 20 (18) | 0.32 |

# Supplementary Table 5. Specific management in ICU

|  | **Global population**  **(n = 185)** | **Good neurological outcome**  **(GOSE 5-8, n = 74)** | **Poor neurological outcome**  **(GOSE 1-4, n = 111)** | **p-value** |
| --- | --- | --- | --- | --- |
| **Osmotherapy.**  **n (%)** | 90 (63) | 37 (50) | 53 (48) | 0.68 |
| **ICP monitoring.**  **n (%)** | 125 (87) | 51 (69) | 74 (67) | 0.35 |
| **EVD placement.**  **n (%)** | 103 (72) | 45 (61) | 58 (52) | 0.83 |
| **Therapeutic hypothermia.**  **n (%)** | 29 (20) | 15 (20) | 14 (13) | 0.37 |
| **Metabolic suppression.**  **n (%)** | 13 (9) | 7 (9) | 6 (5) | 0.60 |
| **Decompressive craniectomy.**  **n (%)** | 19 (11) | 3 (4) | 16 (14) | **0.05** |
| **Temperature >38.5° during ICU stay.**  **n (%)** | 35 (24) | 12 (16) | 23 (21) | 0.31 |
| **Duration of sedation in days.**  **mean (± SD)** | 15.4 (10.6) | 14.7 (8.3) | 16.4 (8.7) | 0.30 |
| **Duration of mechanical ventilation.**  **mean (± SD)** | 22.2 (12.6) | 18.3 (10.8) | 25.1 (13.1) | **0.001** |
| **Tracheostomy.**  **n (%)** | 87 (60) | 43 (58) | 44 (40) | 0.52 |
| **VAP.**  **n (%)** | 107 (75) | 41 (55) | 66 (59) | 0.18 |
| **Infections other than VAP.**  **n (%)** | 55 (38) | 20 (27) | 35 (32) | 0.37 |
| **RRT during ICU stay.**  **n (%)** | 4 (3) | 2 (3) | 2 (2) | 1 |
| **ICU LOS in days.**  **mean (± SD)** | 36.1 (18.1) | 32.2 (14.6) | 38.9 (19.9) | **0.03** |
| **WLST.**  **n (%)** | 54 (32) | 0 | 54 (49) | **<0.0001** |

ICP = intracranial pressure ; EVD = external ventricular drain ; ICU = intensive care unit ; VAP = ventilator-associated pneumoniae ; RRT = renal replacement therapy ; LOS = length of stay ; WLST = withdrawal life-sustaining therapy

# Supplementary Table 6. **AUC according to each scoring system and each rater**

| Score | Rater | IC (95%) |
| --- | --- | --- |
| DAI | Intensivist | 0.65 [0.58- 0.73] |
|  | Radiologist 1 | 0.69 [0.61- 0.76] |
|  | Radiologist 2 | 0.62 [0.55- 0.7] |
| Firsching | Intensivist | 0.68 [0.61- 0.75] |
|  | Radiologist 1 | 0.65 [0.58- 0.71] |
|  | Radiologist 2 | 0.65 [0.57- 0.72] |
| Hamdeh | Intensivist | 0.65 [0.57- 0.72] |
|  | Radiologist 1 | 0.69 [0.61- 0.76] |
|  | Radiologist 2 | 0.6 [0.52- 0.68] |
| Stockholm | Intensivist | 0.66 [0.59- 0.74] |
|  | Radiologist 1 | 0.69 [0.62- 0.76] |
|  | Radiologist 2 | 0.63 [0.56- 0.71] |
| Trondheim | Intensivist | 0.68 [0.61- 0.76] |
|  | Radiologist 1 | 0.7 [0.63- 0.77] |
|  | Radiologist 2 | 0.68 [0.6- 0.75] |

Data are shown as n [IC 95%]

# Supplementary Table 7. ****Results of AUC comparison tests between intensivists and radiologists****

| Score | p-value  (H₀ : Intensivist = Radiol.1) | p-value  (H₀ : Intensivist = Radiol.2) | p-value  (H₀ : Radiol.1 = Radiol.2) |
| --- | --- | --- | --- |
| DAI | 0.39 | 0.32 | 0.12 |
| Firsching | 0.46 | 0.29 | 0.94 |
| Hamdeh | 0.33 | 0.14 | 0.07 |
| Stockholm | 0.51 | 0.33 | 0.21 |
| Trondheim | 0.72 | 0.76 | 0.56 |

# Supplementary Table 8. ****Sensitivity, specificity, PPV, and NPV analyses for each scoring system according to each rater****

|  | Intensivist | | | | | Radiologist 1 | | | | Radiologist 2 | | | | |  |
| --- | --- | --- | --- | --- | --- | --- | --- | --- | --- | --- | --- | --- | --- | --- | --- |
| Scores | Sensitivity | Specificity | PPV | NPV | Sensitivity | | Specificity | PPV | NPV | | Sensitivity | Specificity | PPV | NPV | |
| DAI |  |  |  |  |  | |  |  |  | |  |  |  |  | |
| Grade 1 | 1  [0.97- 1] | 0  [0- 0.05] | 0.6  [0.53- 0.67] | - | 1  [0.97- 1] | | 0  [0- 0.05] | 0.6  [0.53- 0.67] | - | | 1  [0.97- 1] | 0  [0- 0.05] | 0.6  [0.53- 0.67] | - | |
| Grade 2 | 0.89  [0.82- 0.94] | 0.28  [0.19- 0.4] | 0.65  [0.57- 0.72] | 0.64  [0.47- 0.78] | 0.75  [0.66- 0.82] | | 0.58  [0.47- 0.69] | 0.73  [0.64- 0.8] | 0.61  [0.49- 0.71] | | 0.86  [0.78- 0.91] | 0.35  [0.25- 0.46] | 0.66  [0.58- 0.74] | 0.62  [0.47- 0.75] | |
| Grade 3 | 0.67  [0.57- 0.75] | 0.61  [0.49- 0.71] | 0.72  [0.62- 0.8] | 0.55  [0.44- 0.65] | 0.47  [0.38- 0.56] | | 0.8  [0.69- 0.87] | 0.78  [0.66- 0.86] | 0.5  [0.41- 0.59] | | 0.7  [0.61- 0.78] | 0.51  [0.4- 0.62] | 0.68  [0.59- 0.76] | 0.54  [0.42- 0.65] | |
| Firsching | | | | | | | | | | | | | | |  |
| Grade 1 | 1  [0.97- 1] | 0  [0- 0.05] | 0.6  [0.53- 0.67] | - | 1  [0.97- 1] | | 0  [0- 0.05] | 0.6  [0.53- 0.67] | - | | 1  [0.97- 1] | 0  [0- 0.05] | 0.6  [0.53- 0.67] | - | |
| Grade 2 | 0.67  [0.57- 0.75] | 0.61  [0.49- 0.71] | 0.72  [0.62- 0.8] | 0.55  [0.44- 0.65] | 0.47  [0.38- 0.56] | | 0.8  [0.69- 0.87] | 0.78  [0.66- 0.86] | 0.5  [0.41- 0.59] | | 0.7  [0.61- 0.78] | 0.51  [0.4- 0.62] | 0.68  [0.59- 0.76] | 0.54  [0.42- 0.65] | |
| Grade 3 | 0.46  [0.37- 0.55] | 0.82  [0.72- 0.89] | 0.8  [0.68- 0.88] | 0.5  [0.42- 0.59] | 0.26  [0.19- 0.35] | | 0.95  [0.87- 0.98] | 0.88  [0.73- 0.95] | 0.46  [0.38- 0.54] | | 0.32  [0.24- 0.42] | 0.86  [0.77- 0.92] | 0.78  [0.64- 0.88] | 0.46  [0.38- 0.54] | |
| Grade 4 | 0.16  [0.11- 0.24] | 0.97  [0.91- 0.99] | 0.9  [0.7- 0.97] | 0.44  [0.36- 0.51] | 0.13  [0.07- 0.2] | | 0.99  [0.93- 1] | 0.93  [0.7- 0.99] | 0.43  [0.36- 0.5] | | 0.15  [0.1- 0.23] | 0.97  [0.91- 0.99] | 0.89  [0.69- 0.97] | 0.43  [0.36- 0.51] | |
| Hamdeh | | | | | | | | | | | | | | |  |
| Grade 1 | 1  [0.97- 1] | 0  [0- 0.05] | 0.6  [0.53- 0.67] | - | 1  [0.97- 1] | | 0  [0- 0.05] | 0.6  [0.53- 0.67] | - | | 1  [0.97- 1] | 0  [0- 0.05] | 0.6  [0.53- 0.67] | - | |
| Grade 2 | 0.89  [0.82- 0.94] | 0.28  [0.19- 0.4] | 0.65  [0.57- 0.72] | 0.64  [0.47- 0.78] | 0.75  [0.66- 0.82] | | 0.58  [0.47- 0.69] | 0.73  [0.64- 0.8] | 0.61  [0.49- 0.71] | | 0.86  [0.78- 0.91] | 0.35  [0.25- 0.46] | 0.66  [0.58- 0.74] | 0.62  [0.47- 0.75] | |
| Grade 3 | 0.67  [0.57- 0.75] | 0.61  [0.49- 0.71] | 0.72  [0.62- 0.8] | 0.55  [0.44- 0.65] | 0.47  [0.38- 0.56] | | 0.8  [0.69- 0.87] | 0.78  [0.66- 0.86] | 0.5  [0.41- 0.59] | | 0.7  [0.61- 0.78] | 0.51  [0.4- 0.62] | 0.68  [0.59- 0.76] | 0.54  [0.42- 0.65] | |
| Grade 4 | 0.57  [0.47- 0.66] | 0.65  [0.54- 0.75] | 0.71  [0.61- 0.79] | 0.5  [0.4- 0.6] | 0.23  [0.17- 0.32] | | 0.89  [0.8- 0.94] | 0.76  [0.6- 0.88] | 0.44  [0.36- 0.52] | | 0.56  [0.47- 0.65] | 0.55  [0.44- 0.66] | 0.65  [0.55- 0.74] | 0.46  [0.36- 0.56] | |
| Stockholm | | | | | | | | | | | | | | |  |
| Grade 1 | 1  [0.97- 1] | 0  [0- 0.05] | 0.6  [0.53- 0.67] | - | 1  [0.97- 1] | | 0  [0- 0.05] | 0.6  [0.53- 0.67] | - | | 1  [0.97- 1] | 0  [0- 0.05] | 0.6  [0.53- 0.67] | - | |
| Grade 2 | 0.9  [0.84- 0.95] | 0.27  [0.18- 0.38] | 0.65  [0.57- 0.72] | 0.67  [0.49- 0.81] | 0.76  [0.67- 0.83] | | 0.55  [0.44- 0.66] | 0.72  [0.63- 0.79] | 0.6  [0.48- 0.71] | | 0.87  [0.8- 0.92] | 0.34  [0.24- 0.45] | 0.66  [0.58- 0.74] | 0.64  [0.48- 0.77] | |
| Grade 3 | 0.6  [0.53- 0.71] | 0.61  [0.49- 0.71] | 0.7  [0.61- 0.79] | 0.52  [0.41- 0.62] | 0.41  [0.32- 0.5] | | 0.82  [0.72- 0.89] | 0.78  [0.65- 0.86] | 0.48  [0.4- 0.57] | | 0.64  [0.55- 0.72] | 0.53  [0.41- 0.64] | 0.67  [0.58- 0.75] | 0.49  [0.39- 0.6] | |
| Grade 4 | 0.16  [0.11- 0.24] | 0.97  [0.91- 0.99] | 0.9  [0.7- 0.97] | 0.44  [0.36- 0.51] | 0.14  [0.08- 0.21] | | 0.99  [0.93- 1] | 0.94  [0.72- 0.99] | 0.43  [0.36- 0.51] | | 0.14  [0.08- 0.21] | 0.97  [0.91- 0.99] | 0.88  [0.66- 0.97] | 0.43  [0.36- 0.5] | |
| Trondheim | | | | | | | | | | | | | | |  |
| Grade 1 | 1  [0.97- 1] | 0  [0- 0.05] | 0.6  [0.53- 0.67] | - | 1  [0.97- 1] | | 0  [0- 0.05] | 0.6  [0.53- 0.67] | - | | 1  [0.97- 1] | 0  [0- 0.05] | 0.6  [0.53- 0.67] | - | |
| Grade 2 | 0.91  [0.84- 0.95] | 0.24  [0.16- 0.35] | 0.64  [0.57- 0.71] | 0.64  [0.46- 0.79] | 0.76  [0.67- 0.83] | | 0.55  [0.44- 0.66] | 0.72  [0.63- 0.79] | 0.6  [0.48- 0.71] | | 0.87  [0.8- 0.92] | 0.34  [0.24- 0.45] | 0.66  [0.58- 0.74] | 0.64  [0.48- 0.77] | |
| Grade 3 | 0.73  [0.64- 0.8] | 0.49  [0.38- 0.6] | 0.68  [0.59- 0.76] | 0.55  [0.43- 0.66] | 0.49  [0.4- 0.58] | | 0.77  [0.66- 0.85] | 0.76  [0.65- 0.84] | 0.5  [0.41- 0.59] | | 0.76  [0.67- 0.83] | 0.49  [0.38- 0.6] | 0.69  [0.6- 0.76] | 0.57  [0.45- 0.69] | |
| Grade 4 | 0.5  [0.41- 0.6] | 0.8  [0.69- 0.87] | 0.79  [0.68- 0.87] | 0.52  [0.43- 0.61] | 0.3  [0.22- 0.39] | | 0.95  [0.87- 0.98] | 0.89  [0.75- 0.96] | 0.47  [0.39- 0.55] | | 0.35  [0.27- 0.44] | 0.86  [0.77- 0.92] | 0.8  [0.66- 0.89] | 0.47  [0.39- 0.55] | |
| Grade 5 | 0.16  [0.11- 0.24] | 0.97  [0.91- 0.99] | 0.9  [0.7- 0.97] | 0.44  [0.36- 0.51] | 0.13  [0.07- 0.2] | | 0.99  [0.93- 1] | 0.93  [0.7- 0.99] | 0.43  [0.36- 0.5] | | 0.14  [0.09- 0.22] | 0.97  [0.91- 0.99] | 0.89  [0.67- 0.97] | 0.43  [0.36- 0.51] | |

*Data are shown as n [95% IC]*

# Supplementary Figure 2. ****Distribution of GOSE scores by grade and by rater for each radiological scoring system****


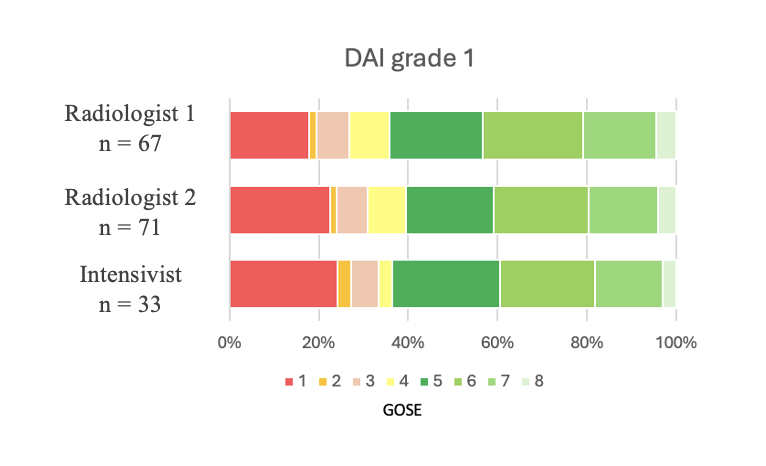


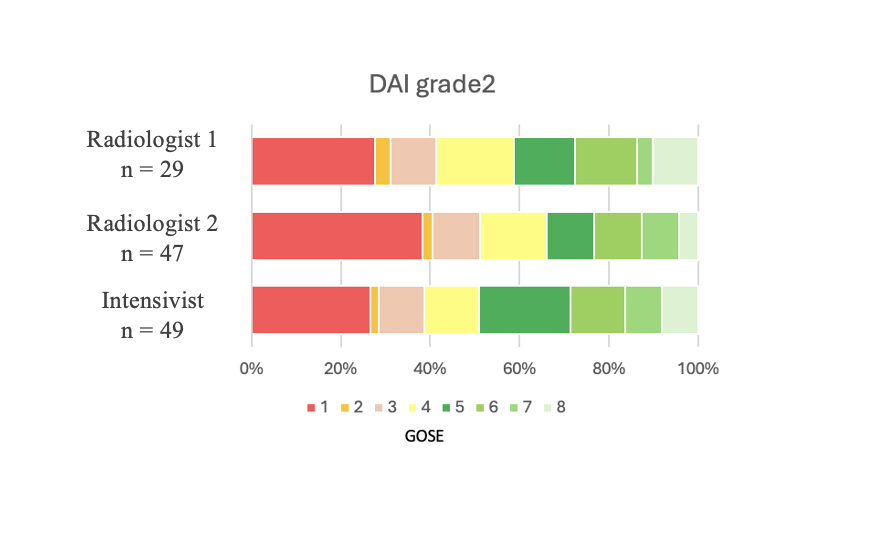


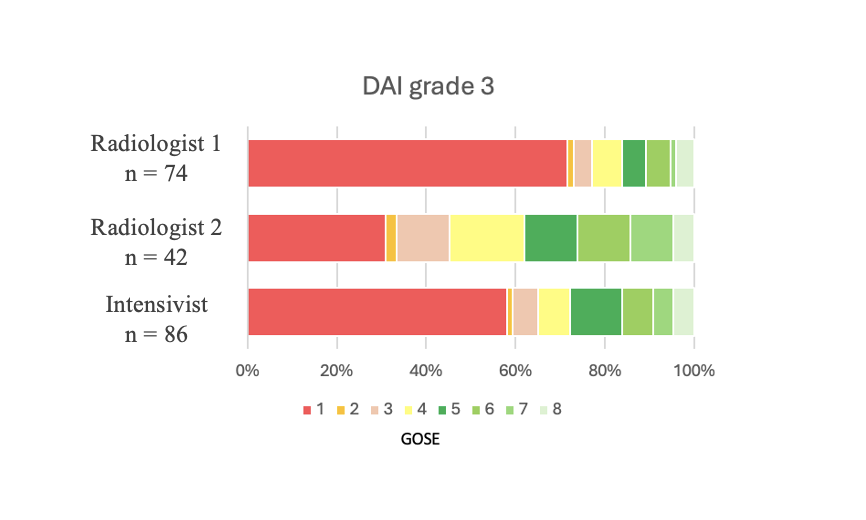


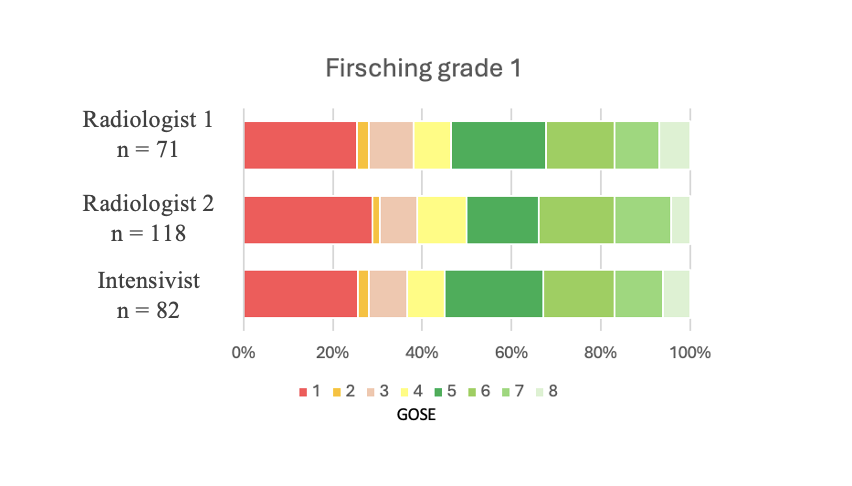


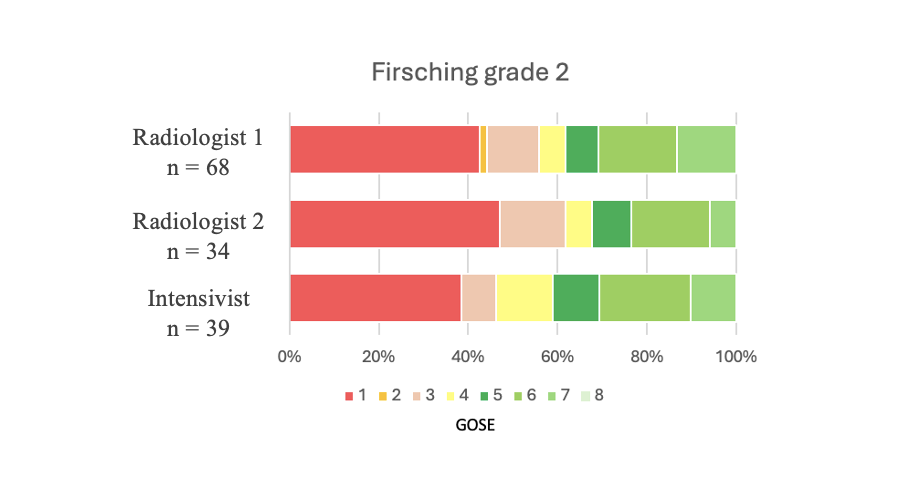


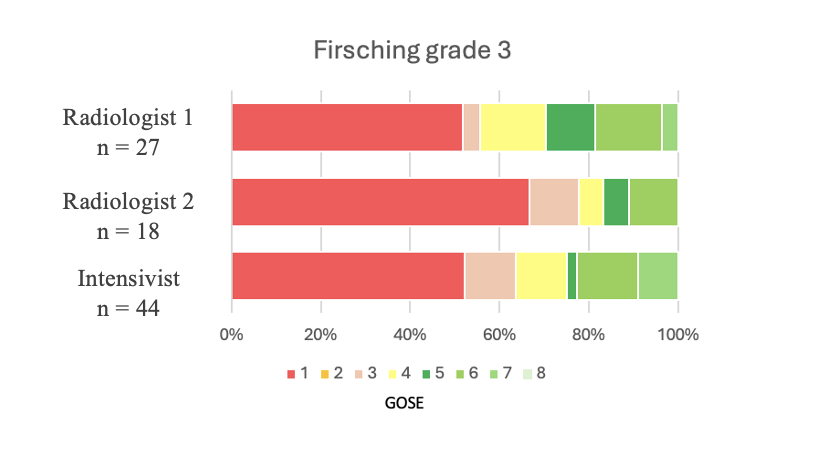


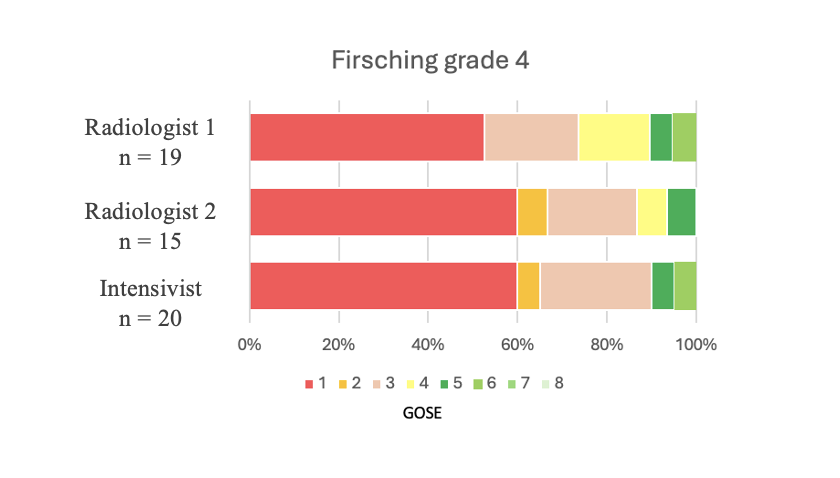


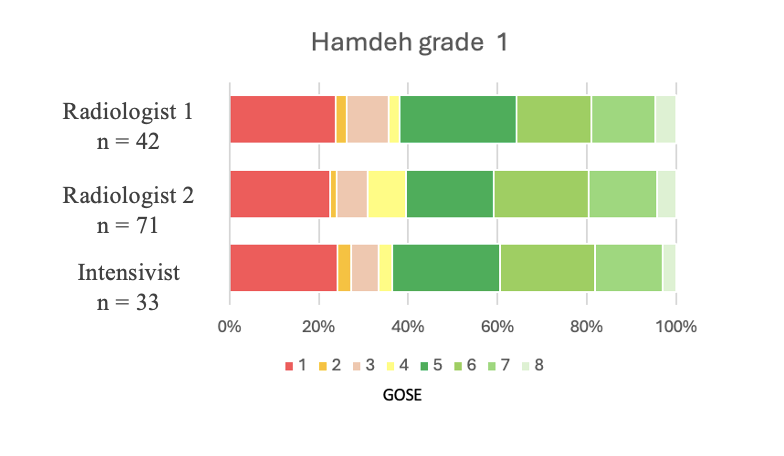


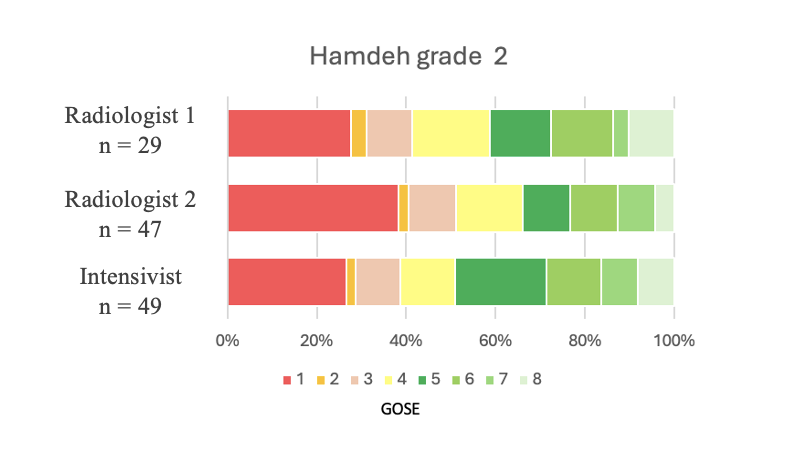


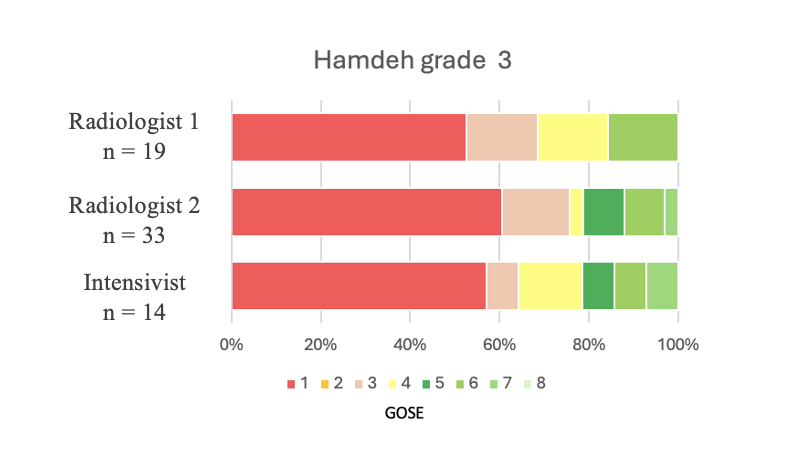


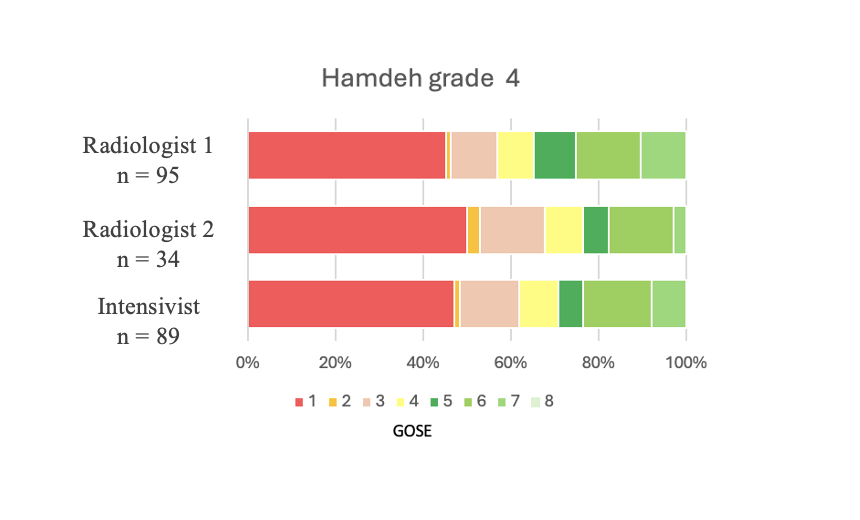


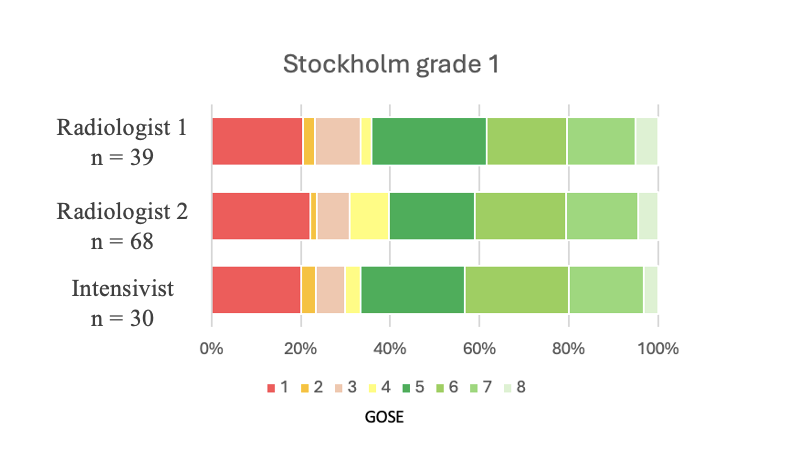


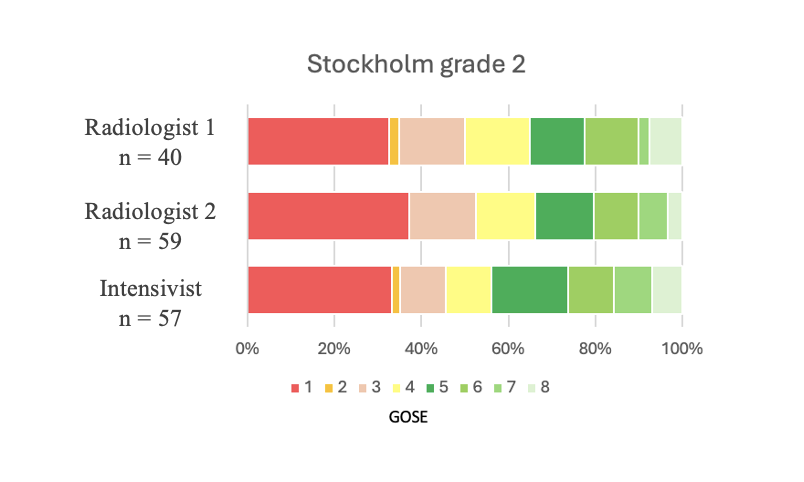


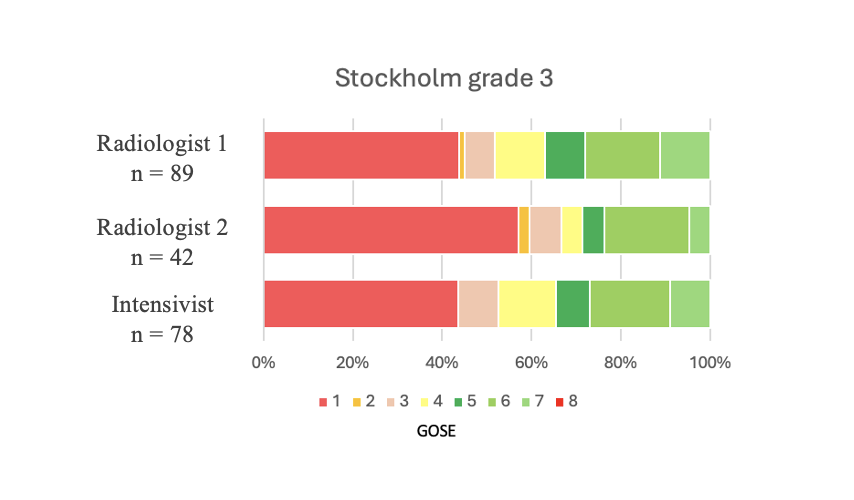


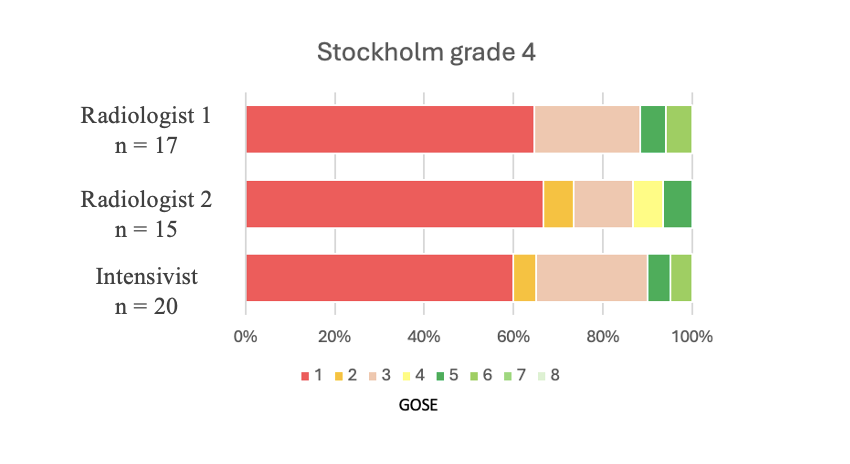


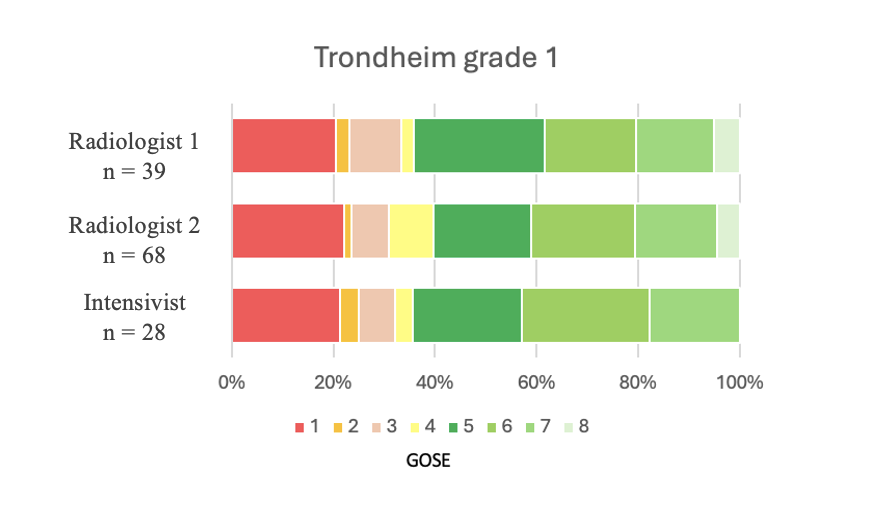


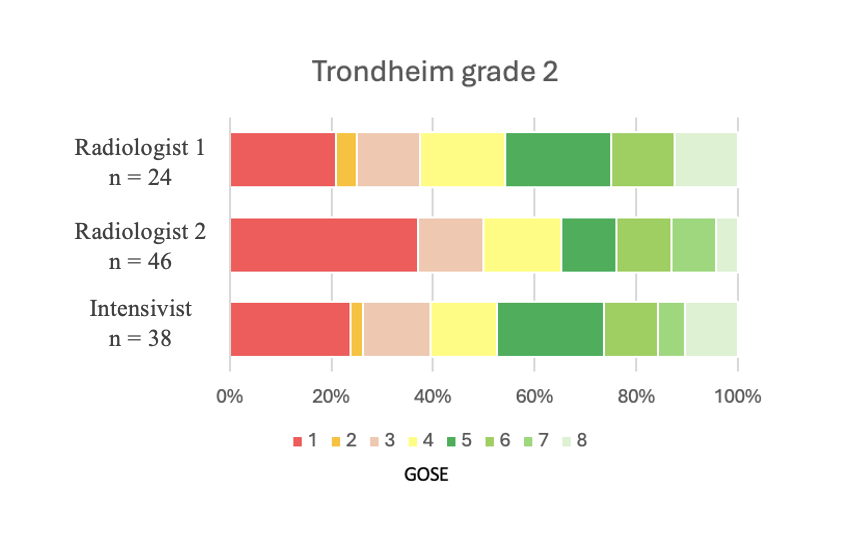


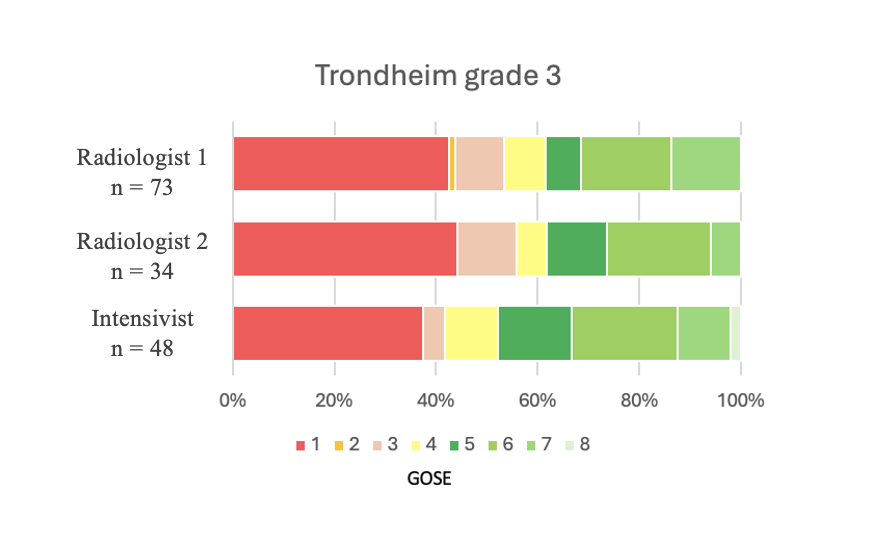


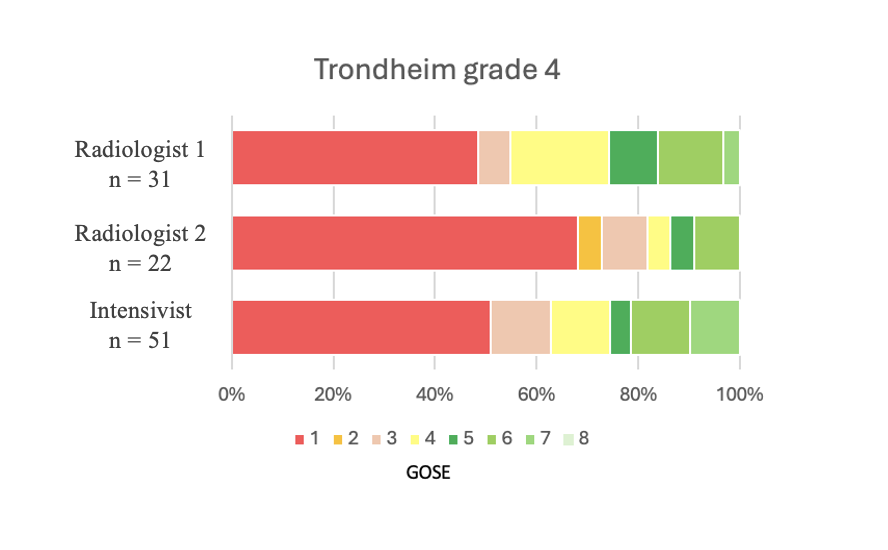


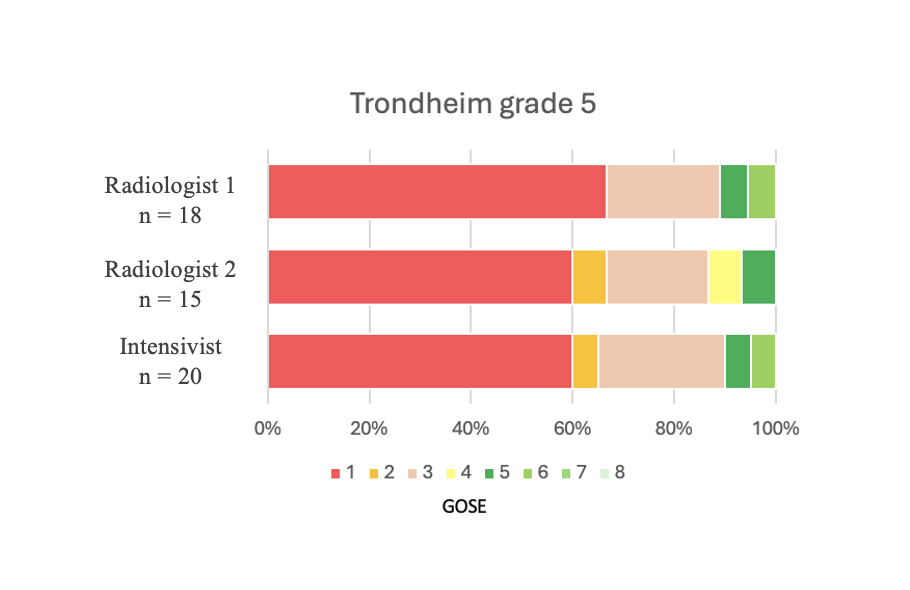


# Supplementary Table 9. ****Median GOSE values for each scoring system and each grade according to each rater****

|  |  | Rater |  |  | p-values |  |
| --- | --- | --- | --- | --- | --- | --- |
| Score | Intensivist | Radiologist 1 | Radiologist 2 | p-value  (H₀ : Intensivist = Radiol.1) | p-value  (H₀ : Intensivist = Radiol.2) | p-value  (H₀ : Radiol.1 = Radiol.2) |
| DAI |  |  |  |  |  |  |
| Grade 1 | 5 [2–6] | 5 [3–6] | 5 [2.25–6] | 0.898 | 0.913 | 0.783 |
| Grade 2 | 4 [1–6] | 3 [1–5] | 4 [1–6] | 0.185 | 0.761 | 0.383 |
| Grade 3 | 3 [1–5] | 1 [1–4] | 3 [1–5] | 0.245 | 0.667 | 0.119 |
| Firsching |  |  |  |  |  |  |
| Grade 1 | 5 [1.25–6] | 4.5 [1–6] | 5 [1.5–6] | 0.71 | 0.942 | 0.78 |
| Grade 2 | 4 [1–6] | 3 [1–5] | 3 [1–6] | 0.331 | 0.741 | 0.478 |
| Grade 3 | 1 [1–4.25] | 1 [1–3] | 1.5 [1–4.25] | 0.239 | 0.942 | 0.255 |
| Grade 4 | 1 [1–3] | 1 [1–3] | 1 [1–3.5] | 1 | 0.463 | 0.489 |
| Hamdeh |  |  |  |  |  |  |
| Grade 1 | 5 [2–6] | 5 [3–6] | 5 [2.25–6] | 0.898 | 0.945 | 0.78 |
| Grade 2 | 4 [1–6] | 3 [1–5] | 4 [1–6] | 0.182 | 0.761 | 0.38 |
| Grade 3 | 1 [1–4] | 1 [1–3] | 1 [1–4] | 0.743 | 1 | 0.65 |
| Grade 4 | 3 [1–5] | 1.5 [1–4] | 3 [1–5.5] | 0.549 | 0.61 | 0.33 |
| Stockholm |  |  |  |  |  |  |
| Grade 1 | 5 [3–6] | 5 [3–6] | 5 [3–6] | 0.823 | 0.844 | 0.995 |
| Grade 2 | 4 [1–6] | 3 [1–5] | 3.5 [1–5] | 0.379 | 0.658 | 0.728 |
| Grade 3 | 3 [1–6] | 1 [1–5] | 3 [1–6] | 0.193 | 0.873 | 0.156 |
| Grade 4 | 1 [1–3] | 1 [1–3] | 1 [1–3] | 0.682 | 0.902 | 0.84 |
| Trondheim |  |  |  |  |  |  |
| Grade 1 | 5 [2.75–6] | 5 [3–6] | 5 [3–6] | 0.984 | 0.99 | 0.995 |
| Grade 2 | 4 [2.25–5.75] | 4 [1–6] | 4 [2.75–5.25] | 0.25 | 0.988 | 0.294 |
| Grade 3 | 4 [1–6] | 1.5 [1–4] | 3 [1–6] | 0.339 | 0.515 | 0.671 |
| Grade 4 | 1 [1–4.5] | 1 [1–4.75] | 3 [1–4.5] | 0.149 | 0.815 | 0.107 |
| Grade 5 | 1 [1–3] | 1 [1–3] | 1 [1–3] | 0.917 | 0.798 | 0.983 |

***P-values were obtained using the Wilcoxon test. Results are presented as median [IQR]***

# References

1. Adams JH, Doyle D, Ford I, Gennarelli TA, Graham DI, Mclellan DR. Diffuse axonal injury in head injury: Definition, diagnosis and grading. Histopathology. juill 1989;15(1):49‑59.

2. Woischneck D, Klein S, Rei�berg S, D�hring W, Peters B, Firsching R. Classification of Severe Head Injury Based on Magnetic Resonance Imaging. Acta Neurochir (Wien). 15 avr 2001;143(3):263‑71.

3. Abu Hamdeh S, Marklund N, Lannsjö M, Howells T, Raininko R, Wikström J, et al. Extended Anatomical Grading in Diffuse Axonal Injury Using MRI: Hemorrhagic Lesions in the Substantia Nigra and Mesencephalic Tegmentum Indicate Poor Long-Term Outcome. J Neurotrauma. 15 janv 2017;34(2):341‑52.

4. Tjerkaski J, Nyström H, Raj R, Lindblad C, Bellander BM, Nelson DW, et al. Extended Analysis of Axonal Injuries Detected Using Magnetic Resonance Imaging in Critically Ill Traumatic Brain Injury Patients. J Neurotrauma. 1 janv 2022;39(1‑2):58‑66.

5. Moen KG, Flusund AMH, Moe HK, Andelic N, Skandsen T, Håberg A, et al. The prognostic importance of traumatic axonal injury on early MRI: the Trondheim TAI-MRI grading and quantitative models. Eur Radiol. 19 juin 2024;34(12):8015‑29.
